# Taurolidine cooperates with antineoplastic drugs in neuroblastoma cells

**DOI:** 10.18632/genesandcancer.36

**Published:** 2014-11

**Authors:** Georg Eschenburg, Christian Luckert, Konrad Reinshagen, Robert Bergholz

**Affiliations:** ^1^ Department of Pediatric Surgery, University Medical Center Hamburg-Eppendorf, Hamburg, Germany

**Keywords:** Neuroblastoma, Apoptosis, Vincristine, Doxorubicin, Experimental Therapies

## Abstract

Neuroblastoma is the most common extracranial tumor in childhood. Outcome of stage 4 disease remains poor and the development of novel therapeutic approaches is thus urgently needed. Taurolidine (TRD), originally invented to avoid catheter infections, has shown to exhibit antineoplastic activity in various cancers. The growth of neuroblastoma cell lines is inhibited by TRD as recently demonstrated. Further analysis disclosed a significant negative growth effect of TRD on the four neuroblastoma cell lines SH-EP TET21N, SK-N-AS, SK-N-BE(2)-M17 and SK-N-SH. Detected IC_50_ (51-274 μM; 48 h) are promising and correspond to clinically-achievable plasma levels. Apoptosis was induced (76-86%; 48 h) in a time-dependent manner mediated by a simultaneous activation of the intrinsic and extrinsic pathways. This was confirmed by cleavage of caspases -3, -8 and -9 and abrogation of apoptosis by pan-caspase inhibition. Application of TRD resulted in a significant enhancement of cytotoxic drugs vincristine/doxorubicin (2/3 of 4 cell lines) making TRD a promising candidate to be included in neuroblastoma therapy regimens in the future.

## INTRODUCTION

Neuroblastoma, originating from sympathetic nervous tissue, is the most common extracranial tumor during childhood. Due to significant improvements in therapy in the recent years 5-year survival rates for non-high risk disease are good (>90%) [1]. However, stage 4 disease with distant organ metastases is highly common at diagnosis.

At the same time, outcome remains poor despite primary surgery and chemotherapy as well as intensive treatment protocols including megatherapy followed by blood stem cell transplantation, differentiation therapy, or immunotherapy [2].

Therefore, the development of novel therapeutic approaches for the treatment of neuroblastoma is one of the main objectives in pediatric oncology [3, 4].

The drug taurolidine [bis(1,1-dioxoperhydro-1,2,4-thiadiazinyl-4)methane] (TRD) is derived from the aminosulfone acid taurine that is able to protect mammalian tissue from oxidant-induced injury [5]. It was originally synthesized by Geistlich-Pharma. TRD was initially used for its antiinflammatory and antimicrobial properties in the treatment of surgical and wound infections as well as for preventing infections by central venous catheters [6-8].

In recent years, substantial evidence was presented for antineoplastic activities of TRD. Cell growth inhibition and induction of apoptosis was achieved *in vitro* and *in vivo* using animal models in such different tumors as bladder carcinoma [9], colon cancer [10-12], epithelioid cell sarcoma [13], esophageal cancer [14], fibrosarcoma [10, 15], gallbladder cancer [16], glioblastoma [17, 18], leiomyosarcoma [13], lung cancer [19], mesothelioma [20], melanoma [21, 22], osteosarcoma [23], ovarian cancer [19], pancreas carcinoma [10], prostate cancer [24] and rhabdomyosarcoma [13].

In neuroblastoma TRD significantly inhibited the cell growth of cell lines SK-N-BE(2)-M17 and SK-N-SH [25]. However, the underlying mechanisms of TRDs mode of action in neuroblastoma cells are unknown so far.

In the extrinsic pathway of apoptosis binding of specific ligands like TNF-α, TRAIL or FASL to so called death receptors on the cell surface initiates the cytosolic formation of complex II containing caspase-8 leading to its activation and caspase-3 subsequently [26].

The intrinsic apoptosis pathway is activated by a plethora of stimuli like viral infections, radiation or treatment with cytotoxic drugs used in chemotherapy [27]. In consequence, processes in the mitochondria evoke a decrease in the mitochondrial membrane potential (MMP), a release of cytochrome C and formation of a complex called apoptosome containing apoptotic protease activating factor 1 (Apaf-1) and caspase-9. The latter is cleaved and in turn activates the effector caspase-3 thereby initiating processes cumulating in apoptosis.

Promising results were obtained with TRD in patients as well. A comprehensive clinical evaluation of TRD is nevertheless missing so far. In one patient with gastric cancer histological remission of the tumor growth was reached. The patient however died from myocardial infarction after occurrence of primary urothelial carcinoma [28]. In stage IV melanoma patients TRD enhanced the tolerability of high-dose interleukin 2 [29].

In two glioblastoma patients TRD was able to significantly improve the quality of life with partial remission of tumor burden [30]. No relevant toxicity was detectable in patients therefore making TRD a promising candidate for further research [28, 31].

Mechanistic analysis of TRD-mediated cell death in cancer cells revealed apoptosis as the main mode of action. Dependent on cell type and experimental setup decrease in mitochondrial membrane potential, cytochrome C release and cleavage of caspase-9 was detected characteristic for the activation of the intrinsic pathway [14, 22, 24, 32, 33]. An activation of the death receptor initiated extrinsic pathway with activation of caspase-8 was observed as well [17, 22, 33].

In the current study we, for the first time, present detailed evidence that TRD significantly induces apoptosis via activating the intrinsic and extrinsic pathways in different neuroblastoma cell lines. TRD cooperates with the cytotoxic drugs doxorubicin and vincristine that are currently used in therapy regimens for neuroblastoma making TRD a promising novel candidate for treatment of this malicious and defying childhood disease.

## RESULTS

### Inhibition of Cellular Growth by Taurolidine in Neuroblastoma Cell Lines

**Figure 1:**
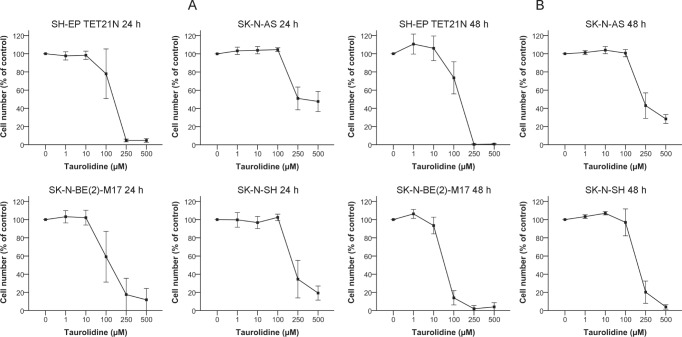
Inhibition of Cell Growth in Neuroblastoma Cell Lines by Taurolidine Cells were treated with the indicated concentrations of TRD and cell growth was determined after 24 h (A) or 48 h (B) using WST-1. Growth of povidone-treated cells was defined as 100 %. Values represent the mean ± SD of three to four independent experiments.

We first evaluated the growth of human neuroblastoma cell lines (n=4) in the presence of increasing amounts (0-500 μM) of taurolidine (TRD). Cellular growth was inhibited by TRD in all cell lines. Decrease in growth 24 h following treatment initiation was seen with a minimal dose of 100 μM TRD (figure 1A). The cell line SK-N-BE(2)-M17 was most sensitive with an IC_50_ of 126 μM, the IC_50_ of the other cell lines varied between 152-353 μM (table 1).

**Table 1:** Growth of Neuroblastoma Cell Lines is Inhibited by Taurolidine in a Concentration-dependent Manner IC_50_, IC_75_ and IC_90_ values for dose-effect curves were determined 24 h and 48 h following treatment. n.d. = not determined.

	Taurolidine (μM)					
	IC_50_		IC_75_		IC_90_	
	24 h	48 h	24 h	48 h	24 h	48 h
**SH-EP TET21N**	152	151	196	203	222	235
**SK-N-AS**	353	274	n.d.	522	n.d.	n.d.
**SK-N-BE(2)-M17**	126	51	210	80	284	102
**SK-N-SH**	224	186	275	225	310	248

Analysis of cellular growth after 48 h of treatment revealed a stronger impact of TRD in comparison to the 24 h time point as expected (figure 1B). IC_50_ values were between 51 μM (SK-N-BE(2)-M17) and 274 μM (SK-N-AS) at 48 h (table 1).

### Neuroblastoma Cells Undergo TRD-induced Apoptosis

In order to evaluate if apoptosis is causal for the TRD-induced decrease of cell growth neuroblastoma cells were analyzed using flow cytometry.

**Figure 2:**
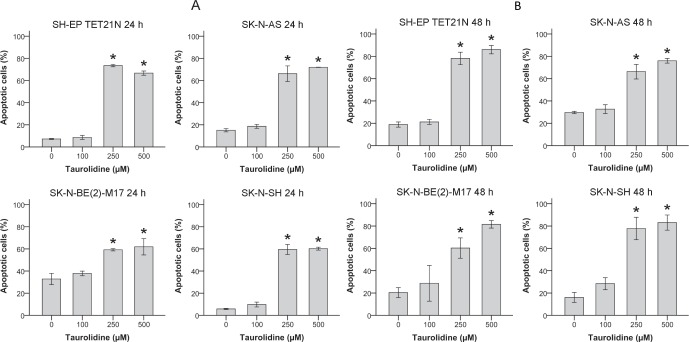
Apoptosis Induction in Neuroblastoma Cell Lines by Taurolidine Apoptosis induced by TRD was analyzed over time. Cells were treated with the indicated concentrations of TRD and the proportion of apoptotic cells was determined by flow cytometry (Annexin V and PI staining) after 24 h (A) or 48 h (B). Values represent the mean ± SD of three independent experiments, *; p ≤ 0.05.

In all cell lines TRD induced apoptosis characterized by externalization of phosphatidylserine (Annexin V staining) and PI uptake (figures 2A-B). Apoptotic cells significantly increased (p ≤ 0.05) in a time- and concentration-dependent way with a maximum apoptosis of 86% in the cell line SH-EP TET21N (500 μM TRD; 48 h) (figure 2B).

To generate additional evidence for TRD's potential to stimulate the induction of apoptosis in neuroblastoma the cleavage of caspase-3 to its active fragment, a specific feature of apoptosis, was determined [34].

**Figure 3:**
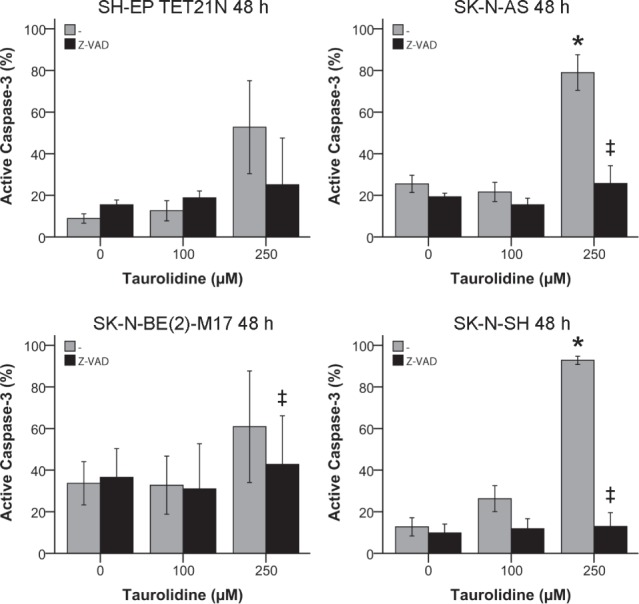
Effect of Taurolidine on Effector Caspase-3 in Neuroblastoma Cell Lines Activation of active caspase-3 was determined by staining with FITC-DEVD-FMK and flow cytometry 48 h after treatment with TRD and 100 μM pan-caspase inhibitor Z-VAD. Values represent the mean ± SD of three independent experiments, * (TRD 0 μM vs. 250 μM) and ‡ (TRD 250 μM +/− Z-VAD); p ≤ 0.05.

Treatment with TRD caused a significant induction of active caspase-3 in a concentration-dependent manner (figure 3). Cleavage of caspase-3 was significantly inhibited (p ≤ 0.05) in three of four cell lines if cells were additionally pre-treated with pan-caspase inhibitor Z-VAD (figure 3) giving evidence that the impact of TRD on apoptosis is mediated by upstream caspases activation.

### Taurolidine-mediated Apoptosis is Abolished by Pan-Caspase-inhibition

For further characterization of the molecular pathways involved in TRD-induced apoptosis specific and pan-caspase inhibition was conducted.

**Figure 4:**
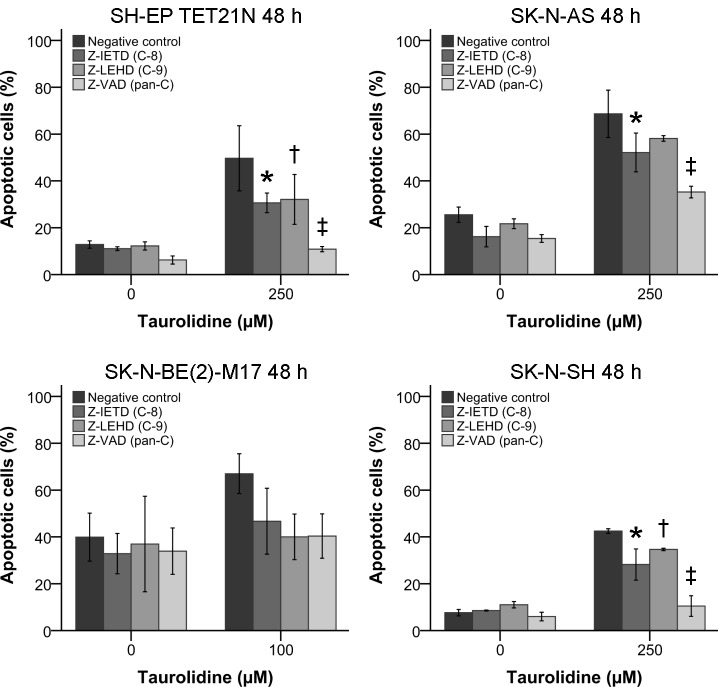
Influence of Caspase-inhibition on Taurolidine-inducedApoptosis Induction Neuroblastoma cells were treated with the indicated concentrations of TRD and 100 μM caspase inhibitors. The proportion of apoptotic cells was determined by flow cytometry and staining with Annexin V and PI 48 h after treatment initiation. Values represent the mean ± SD of three independent experiments, * (TRD 250 μM + NC vs. Z-IETD), † (TRD 250 μM + NC vs. Z-LEHD) and ‡ (TRD 250 μM + NC vs. Z-VAD); p ≤ 0.05. NC = Negative control.

Neuroblastoma cells were treated with TRD for 48 h and apoptosis was quantified by flow cytometry. TRD-induced apoptosis was reduced by inhibition of caspase-8 or caspase-9 in all four neuroblastoma cell lines respectively (figure 4).

The apoptosis reduction was significant (p ≤ 0.05) using caspase-8 inhibitor in three of four cell lines and with caspase-9 inhibitor in two of four cell lines.

Consistent with the former findings pan-caspase inhibition, by irreversibly binding the active site of activated proteases using Z-VAD, significantly (p ≤ 0.05) reduced the effect of TRD to basal levels in three of four cell lines (figures 3 and 4).

### Taurolidine Induces Activation of Intrinsic and Extrinsic Apoptosis Pathways

After relevant inhibition of TRD-mediated apoptosis was achieved by blockade of active caspases-8 and -9 the effects of TRD on the activation of the in- and extrinsic apoptosis pathways was quantified by flow cytometry.

**Figure 5:**
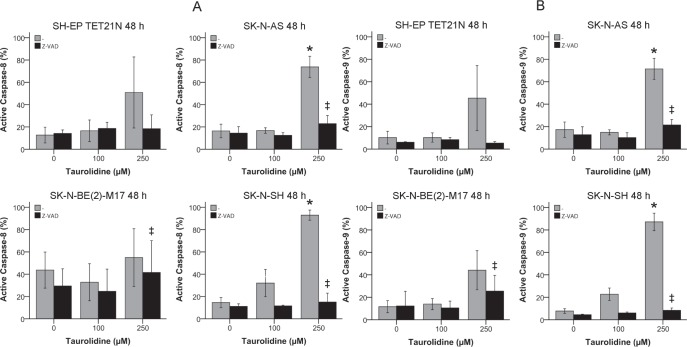
Activation of the Extrinsic and Intrinsic Apoptosis Pathways in Neuroblastoma Cells by Taurolidine Activation of active caspase-8 (A) and caspase-9 (B) was determined by staining with FITC-IETD-FMK (C-8) or FITC-LEHD-FMK (C-9) respectively and flow cytometry 48 h after treatment with TRD and 100 μM pan-caspase inhibitor Z-VAD. Values represent the mean ± SD of three independent experiments, * (TRD 0 μM vs. 250 μM) and ‡ (TRD 250 μM +/− Z-VAD); p ≤ 0.05.

Treatment with TRD significantly induced (p ≤ 0.05) the formation of active caspase-8 and -9 in a concentration-dependent way indicative for extrinsic and intrinsic apoptosis in the cell lines SK-N-AS and SK-N-BE(2)-M17 (figures 5A-B). The activation of both caspases was significantly impeded with Z-VAD pan-caspase inhibitor treatment in all cell lines with the exception of SH-EP TET21N (figures 5A-B).

### Apoptosis Induction of TRD in Combination with Vincristine and Doxorubicin

The vinca alkaloid vincristine and the antracycline doxorubicin are antineoplastic drugs commonly used for neuroblastoma chemotherapy [3]. Thus, it was tested if TRD has the potential to improve the effects of these drugs.

**Figure 6:**
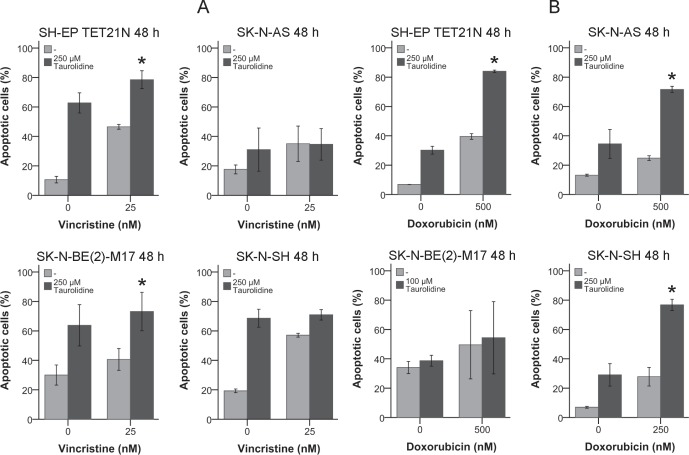
Effect of Taurolidine and Antineoplastic Drugs on Apoptosis Induction in Neuroblastoma Cells Neuroblastoma cells were treated with the indicated concentrations of TRD, vincristine (A)/doxorubicin (B) or a combination of both. Apoptotic cells were determined by Annexin V and PI staining and flow cytometry 48 h post treatment initiation. Values represent the mean ± SD of three independent experiments, *; p ≤ 0.05.

All cell lines were susceptible for 25 nM vincristine with an induction of apoptosis of 35-57% after 48 h (figure 6A). The combination of vincristine with TRD resulted in an apoptosis induction of 35-79% and a significant increase (p ≤ 0.05) of the vincristine effect in the cell lines SH-EP TET21N and SK-N-BE(2)-M17 (figure 6A).

Treatment of neuroblastoma cell lines with 250-500 nM doxorubicin effected an apoptosis induction between 25% and 50% (figure 6B). In all cell lines except SK-N-BE(2)-M17 the combination of doxorubicin with TRD resulted in a significant mutual enhancement (p ≤ 0.05) with an increase of apoptotic cells to 71-84%.

This results show TRD's capability to support the effect of cytotoxic drugs in neuroblastoma cell lines.

## DISCUSSION

Taurolidine (TRD) is an innovative drug in cancer therapy that demonstrated antiproliferative and proapoptotic potential *in vitro* and *in vivo* in many malignancies reaching from bladder to prostate cancer [9, 24].

To further establish the potential use of TRD for neuroblastoma therapy detailed analyses of TRD's impact on regulation of apoptosis and the underlying pathways were conducted.

For the first time we were able to show that treatment of neuroblastoma cell lines with TRD significantly inhibits cellular growth and induces apoptosis (figures 1 + 2). Externalization of phosphatidylserine detected by Annexin V staining significantly increased after TRD-treatment, a specific cellular reaction in early apoptosis [35].

In the same way cells double positive for PI and Annexin V were elevated. As PI uptake can either be a consequence of necrosis or an indication for late stage apoptosis a time-dependent increase of only Annexin V-positive cells supports that apoptosis is the main mode of action in TRD-mediated cell death in neuroblastoma [36].

Furthermore, TRD treatment induced detachment, round shaping, and shrinkage of the cells typical morphological transformations of apoptosis [37].

Along with Annexin V-positivity the cleavage of caspase-3 to its active fragment is a specific marker of apoptosis [34]. In line with this, TRD significantly induced active caspase-3 in neuroblastoma an effect which was also shown in glioma and malignant pleural mesothelioma (figure 3) [33, 38]. Almost complete abrogation of caspase-3 activation by pan-caspase inhibitor Z-VAD indicated that upstream caspases are involved in TRD-mediated apoptosis (figure 3).

Inhibition of caspases-8 and -9 decreased apoptosis induction and pan-caspase blockade reduced apoptosis to almost basal levels. These findings indicate that intrinsic (mitochondrial) and extrinsic (death receptor) apoptosis pathways are involved (figure 4).

An induction of the mitochondrial apoptotic signaling pathway was described for TRD in several different malignancies. In melanoma, TRD led to expression of the proapoptotic Bax and to inhibition of the antiapoptotic Bcl-2 critical mediators of the intrinsic pathway [22]. Bcl-2 was decreased in malignant pleural mesothelioma following TRD-treatment too [33].

The effect of TRD on the intrinsic pathway was further supported by a loss of mitochondrial membrane potential and cytochrome C translocation to the cytosol in glioma, leukemia and prostate cancer [24, 32, 38]. An activation or upregulation of caspase-9 was detected in models of esophageal and prostate cancer [14, 24].

TRD's influence on the extrinsic pathway is less well documented. TRD mediated antitumor effects were postulated as in part being based on an increase in Fas-ligand induced apoptosis in glioblastoma [17]. Upregulation of caspase-8 and the whole TNF-receptor signaling was seen in squamous carcinoma giving thus no direct evidence for pathway activity [14]. In prostate cancer activation of caspase-8 following TRD treatment was described but subsequent to activation of caspase-9 [24]. This indicates a cross-talk between both apoptosis pathways as seen in other diseases [39].

As described, TRD-induced apoptosis in neuroblastoma was dependent on caspases-8 and -9 (figure 4). Accordingly, activated caspases-8 and -9 were significantly induced following TRD exposure in two of four cell lines (figures 5A-B). Significant differences in caspase-dependency of TRD-mediated apoptosis would be expected in neuroblastoma if caspase-8 is activated subsequent to caspase-9 or vice versa [24, 40, 41]. No obvious differences were observed and therefore an independent activation of both caspases through TRD is suggested.

TRD was tested in combination with different cytotoxic drugs in order to evaluate its potential to efficiently supplement classic treatment of neuroblastoma. The antimitotic vinca alkaloid vincristine effectively induced apoptosis in the treated neuroblastoma cell lines (figure 6A). Cytotoxic therapy using vincristine was further and significantly increased in the cell lines SH-EP TET21N and SK-N-BE(2)-M17 (figure 6A).

The effects of the anthracycline doxorubicin with the used doses were only moderate but were significantly amplified in three of four cell lines if it was applicated in company with TRD (figure 6B).

The capacity of TRD to cooperate with additional other drugs used for neuroblastoma therapy would be informative. Relevant insights could thus be obtained if TRD's impact on molecular level could be elucidated and then be correlated to the modes of action of the cytotoxic drugs and the observed effects of the combination treatment.

In few other cancers TRD was tested along with cytotoxic drugs. In malignant pleural mesothelioma TRD effected a synergistically enhancement of cisplatin [33]. Combination of TRD with a variety of drugs in fibrosarcoma revealed only a synergistic effect for mafosfamide and an unexpected antagonism for doxorubicin and trabectedin [15].

Reasons for these adverse effects of some drug combinations are unknown so far and could be important hurdles for a transfer of TRD to clinical application for cancer treatment.

Using animal cancer models TRD showed a broad spectrum of activity *in vivo*. Malignant mesothelioma, melanoma, ovarian and prostate cancer were efficiently reduced in mice [19, 20, 22, 24].

Comprehensive studies were conducted evaluating TRD's effect on colon cancer in a rat model revealing inhibition of tumor growth and prevention of metastases [42]. In Syrian hamsters TRD was potent in treatment of pancreatic carcinoma [43].

Beside these promising results in different animal systems some findings showed side-effects of TRD in mice and rats. In a murine osteosarcoma model TRD was not only ineffective in tumor reduction but also enhanced the occurrence of metastasis and liver deformations which was associated with a significant mortality of ~40% [44]. In rat bladder carcinoma TRD also promoted tumor growth [9].

Interestingly in both systems TRD was effective *in vitro* and *in vivo* TRD concentrations (500-800 mg/kg) were comparable with other surveys where a mortality of 10% in maximum was observed.

Obviously TRD has a rather small therapeutic index if the negative effects of TRD in some settings are taken into account.

Nevertheless, TRD is a promising novel drug with increasing evidence of remarkable potency against cancer growth in animal models.

In patients TRD was used as palliative option which was well tolerated giving rise to the hope that TRD might be an up-and-coming drug to support treatment and enhance the clinical outcome of cancer [28-31].

Our results in neuroblastoma cell lines are promising as well. The obtained IC_50_ of TRD at 48 h time point were between 51-274 μM and hence in a range that is clinically-achievable. Repeated intravenous application of TRD in humans induced detectable plasma levels of 96 μg/ml (337 μM) [45].

The evaluation of TRD in a murine neuroblastoma system recapitulating the significant effects that we could show *in vitro* is therefore the consequential next experimental step.

## METHODS

### Cell lines and cell culture

Neuroblastoma cell lines SH-EP TET21N, SK-N-AS, SK-N-BE(2)-M17 and SK-N-SH were used. SH-EP TET21N is a conditional, tetracycline-regulated MYCN expression system established in the MYCN non-amplified SH-EP neuroblastoma cell line [46]. Neuroblastoma cells were cultured in RPMI 1640 medium supplemented with 10% fetal bovine serum (both Life Technologies, Darmstadt, Germany) and Penicillin/Streptomycin (10.000 U/ml / 10.000 μg/ml, Biochrom, Berlin, Germany). All cells were cultivated at 37°C, 5% CO_2_-atmosphere and a relative humidity of 95%.

### Chemical compounds, biological reagents and drugs

Taurolidine (Taurolin^®^, Berlin-Chemie AG, Berlin, Germany) working solutions were prepared by dilution with PBS (Life Technologies, Darmstadt, Germany) to designated concentrations. Povidone (Polyvinylpyrrolidone, Sigma-Aldrich, Steinheim, Germany) had a concentration of 5% in the undiluted TRD and was dissolved with PBS to final concentration. Cytostatic drugs were obtained from Sigma-Aldrich (Munich, Germany). Inhibitors targeting caspase-8 (Z-IETD; FMK007) and -9 (Z-LEHD; FMK008) as well as pan-caspase inhibitor (Z-VAD; FMK001) were obtained from R&D (Wiesbaden, Germany).

### Cellular growth assays

Assays were performed with Cell Proliferation Reagent WST-1 (Roche, Grenzach, Germany) according to the manufacturer's protocol. Cells were seeded in phenol red-free culture medium in 96-well plates to adhere overnight. TRD was added to the cells for an incubation period of 24-48 h. To exclude povidone-related effects in TRD-treated cells, it was added to controls in a concentration equally to its concentration in the highest used dosage of TRD. Following incubation with WST-1 for 2 h, absorbance was measured with an ELISA reader. IC _50_, IC_75_ and IC_90_ values were determined using in-house software (Microsoft Excel).

### Detection of apoptosis by flow cytometry

Cells were seeded in cell culture medium in 24-well plates to adhere overnight and then were treated with the indicated reagents for 24 h to 48 h. They were harvested, washed twice with PBS and resuspended in Annexin V binding buffer (10 mM Hepes, 140 mM NaCl, and 0.25 mM CaCl_2_). Apoptosis was detected by Annexin V-FITC (556419; BD Pharmingen, Heidelberg, Germany) and propidium-iodide (PI) (1 mg/ml in ddH_2_ O; Invitrogen, Darmstadt, Germany) staining and flow cytometry.

### Detection of active caspases-3, -8 and -9

Cells were seeded in cell culture medium in 24-well plates to adhere overnight and then were treated with the indicated reagents for 48 h. Cells were harvested, washed, aliquoted into FACS tubes and detection of active caspases was performed using the Green Caspase-3 (PK-CA577-K183-100; PromoKine), Green Caspase-8 (-K188-100) and Green Caspase-9 (-K189-100) Staining Kits. Cells were stained with 1 μl FITC-DEVD-FMK (C-3), FITC-IETD-FMK (C-8) or FITC-LEHD-FMK (C-9) and incubated for 1h at 37°C. Cells were washed twice, resuspended in wash buffer, kept on ice and active caspase expression was measured using flow cytometry.

### Statistical analysis

The amount of vital cells treated with TRD was calculated as percentage to the amount of vital cells in the control group (5% povidone). The software IBM SPSS was used to perform the statistical analysis. Statistical significance of differences between treatment groups was determined using a Student t-test. A p-value ≤ 0.05 was regarded as significant.

## Abbreviations

FACS: fluorescence-activated cell sorting
IC: inhibitory concentration
MMP: mitochondrial membrane potential
PI: propidium-iodide
TRD: taurolidine
